# Preimplantation genetic screening (PGS) still in search of a clinical application: a systematic review

**DOI:** 10.1186/1477-7827-12-22

**Published:** 2014-03-15

**Authors:** Norbert Gleicher, Vitaly A Kushnir, David H Barad

**Affiliations:** 1 The Center for Human Reproduction (CHR), New York, USA; 2 The Foundation for Reproductive Medicine, New York, USA

**Keywords:** Preimplantation genetic diagnosis (PGS), Assisted reproduction (ART), In vitro fertilization (IVF), Trophectoderm biopsy, Blastocyst stage embryo transfer

## Abstract

Only a few years ago the *American Society of Assisted Reproductive Medicine (ASRM)*, the *European Society for Human Reproduction and Embryology (ESHRE*) and the *British Fertility Society* declared preimplantation genetic screening (PGS#1) ineffective in improving in vitro fertilization (IVF) pregnancy rates and in reducing miscarriage rates. A presumably upgraded form of the procedure (PGS#2) has recently been reintroduced, and is here assessed in a systematic review. PGS#2 in comparison to PGS#1 is characterized by: (i) trophectoderm biopsy on day 5/6 embryos in place of day-3 embryo biopsy; and (ii) fluorescence in-situ hybridization (FISH) of limited chromosome numbers is replaced by techniques, allowing aneuploidy assessments of all 24 chromosome pairs. Reviewing the literature, we were unable to identify properly conducted prospective clinical trials in which IVF outcomes were assessed based on “intent to treat”. Whether PGS#2 improves IVF outcomes can, therefore, not be determined. Reassessments of data, alleged to support the efficacy of PGS#2, indeed, suggest the opposite. Like with PGS#1, the introduction of PGS#2 into unrestricted IVF practice again appears premature, and threatens to repeat the PGS#1 experience, when thousands of women experienced reductions in IVF pregnancy chances, while expecting improvements. PGS#2 is an unproven and still experimental procedure, which, until evidence suggests otherwise, should only be offered under study conditions, and with appropriate informed consents.

## Background

In July 2007 Mastenbroek et al. published one of the most important papers in the history of in vitro fertilization (IVF) when they reported that preimplantation genetic screening (PGS) in women of advanced age not only failed to improve but actually diminished ongoing IVF pregnancy and live birth rates [1]. Despite some valid criticisms of the study [2], the historic importance of this paper stems from its effects on clinical practice since its publication reduced the worldwide utilization of PGS in attempts to improve IVF outcomes, as indicated by U.S. [3] and European data from a 10-year ESHRE report, which demonstrated a first decline in PGS numbers [4] in 2007, the year Mastenbroek’s paper appeared in print [1]. For the rest of this communication this initial form of PGS will be described as PGS#1.

Because the basic hypothesis of PGS that the transfer of only euploid embryos should improve IVF outcomes is so attractively logical, up to publication of Mastenbroek’s paper, proponents of PGS#1 had convinced a majority of the worldwide IVF community that it represented a valid approach towards improving IVF outcomes. What PGS#1, however, really accomplished was to reduce pregnancy chances for a significant proportion of women utilizing the procedure, mostly older women, without offering benefits to others [5-7]. PGS#1, thus, achieved the unique notoriety of becoming the first ever widely introduced routine IVF practice, which actually harmed IVF cycle outcomes. By reducing this practice, the 2007 publication by Mastenboek et al. established its historical prominence [1].

Mastenbroek et al., however, were not the first to notice the futility of PGS#1: Earlier, Belgian investigators failed to detect IVF outcome benefits from PGS#1 in three smaller clinical trials [8-10]. Further analyzing the 2004 paper by Staessen et al. [8], we started to suspect that PGS#1 in older study populations might actually reduce IVF pregnancy chances. Our suspicion was raised because the study’s older patient population produced significantly lower embryo numbers for transfer and an almost statistically significant trend towards lower pregnancy rates (P = 0.06) in association with PGS#1.

We, therefore, were not surprised by the 2007 study of Mastenbroek et al. [1]. A paper, outlining our suspicion, and including a reanalysis of the Belgian data, was, however, only accepted (in expedited review) after Mastenbroek’s paper had appeared [11]. Our suspicion that pregnancy chances of older women are actually hurt by the procedure was subsequently repeatedly demonstrated [5-7]. The importance of these age-related findings was reemphasized by the fact that, at least in Europe, the primary indication for PGS#1 was advanced female age [4].

Just a few years later, history appears to repeat itself: A supposedly improved version of PGS (PGS#2), claiming outcome benefits for IVF, is now, once again, being promoted. This reintroduction of PGS in the form of PGS#2 is mostly based on allegedly improved techniques and technologies, with ability to diagnose aneuploidies more accurately.

In a review of PGS#1 we recently reached the conclusion that the failure of PGS#1, likely, was not, as suggested by proponents of PGS#2, the consequence of inadequate PGS#1 techniques and technologies but, primarily, due to incorrect patient selection. We, therefore, cautioned from uncritically accepting the notion that improvements in PGS#1 techniques and technologies, alone, would improve IVF outcomes [12].

We here present a systematic review of published PGS#2 experiences in an attempt to determine whether so far reported results with PGS#2, indeed, do improve clinical pregnancy and delivery rates and/or reduce miscarriage rates in association with IVF.

## Methods

We reviewed the medical literature via PubMed and Medline searches under appropriate keyword and phrases up to year-end 2013. Keywords included < preimplantation genetic diagnosis > and the abbreviating < PGD>; <preimplantation genetic screening > and the abbreviation < PGS>; the phrases < genetic screening with in vitro fertilization/IVF > and < genetic diagnosis with in vitro fertilization/IVF>. We also searched under the phrases < arrays in IVF>, <comparative genomic hybridization in IVF>, <comprehensive chromosome screening in IVF > and <24 chromosome copy analysis in IVF > .

The primary review was performed by one of the authors (N.G.), with the other two authors reviewing selected publications in conjunction with internal reevaluations of published data. The primary review of the literature revealed 93 relevant articles. Amongst those, 39 were chosen as reflective of published data on PGS#2, and as references with relevance to the presentation of context. Five of these references, providing context, were added based on suggestions received during the manuscript review process. It is important to note that all published papers in the English literature addressing PGS#2 during the search years were reviewed. This study presents all published data on PGS#2, independent of our quality assessment. In addition, we reviewed reference lists of reviewed papers for additional appropriate articles.

Since this literature review did not reveal even a single study in which PGS#2 was evaluated based on “intent to treat” (i.e., with reference point IVF cycle start), this review cannot report a metaanalysis of reported data. While this finding, alone, suggests that so far available PGS#2 data are insufficient to support the uncontrolled utilization of PGS#2 as a routine feature of IVF practice, our research did discover published studies, claiming to be represent results of clinical trials demonstrating outcome benefits for IVF following utilization of PGS#2. Since our evaluation of these studies disagreed with the authors’ conclusions, two such studies are here discussed in detail.

To make sure no relevant studies were overlooked, we also reviewed all currently registered PGS trials, cross-referencing the names of principal investigators under above noted key words and phrases in our literature search.

## Results

As noted above, we were unable to find even one appropriately performed prospectively randomized clinical trial, which assessed IVF outcomes with use of PGS#2 based on “intent to treat.” Also discussed in more detail below, this prohibits a statistically valid assessment of PGS#2 in its utility to improve IVF outcomes by meta-analysis. We made this observation already in 2012, noting at the time that such studies would only unlikely appear in the literature in the foreseeable future since among all formally registered ongoing clinical trials on the subject, none appeared to use properly designed statistical methodologies, including analysis of IVF outcomes by “intent to treat” [12].

Here obtained results, therefore, are not necessarily surprising. Only one then registered clinical trial has, since, been completed in 2012, has been reported, and will be discussed in detail below. The statistical methodologies utilized in this trial fully confirmed our then voiced concerns [12]. Absence of properly designed clinical trials is especially noteworthy as patient surveys suggest willingness to participate in such trials [13].

Though results of this systemic review, thus, have failed to demonstrate adequate supportive evidence for the clinical utilization PGS#2 outside of experimental frameworks, this review, nevertheless, offers additional potentially important insights into the current practice of PGS#2, presented below in the discussion section of this manuscript.

## Discussion

Two studies, receiving extraordinary attention for claiming outcome benefits for PGS#2, appeared in print during 2013. In addition, at least one editorial opinion was supportive of PGS#2 [14], though others [15] and we [16] disagreed. In this discussion section we, therefore, pay special attention to these two studies, partially reassessing their data, as reported by the investigators.

### How PGS#2 is misrepresented

To understand the importance of outcome calculations in assessing the efficacy of PGS#2 based on “intent to treat”, differences between PGS#1 and PGS#2 have to be clearly understood. A few major technical and methodological changes differentiate between the two:

(i) PGS#2 relies on trophectoderm biopsy of embryos on days 5–6 after fertilization, while PGS#1 relied on day-3 blastomere biopsy. One important difference between these two techniques, therefore, lies in trophectoderm biopsy mandating embryo culture to days 5/6. Unless viable embryos reach blastocyst stage, patients are not even given a chance of embryo biopsy and chromosomal analysis in association with PGS#2. Not all embryos, however, reach blastocyst stage. Especially embryos from older women and younger females with prematurely low ovarian reserve often do not. Only better prognosis patients do culture successfully *in vitro* up to days 5/6. This fact is, indeed, so well known that some IVF centers utilize culture to blastocyst stage as a method of embryo selection, allowing the “fittest” to survive.

Trophectoderm biopsy in association with PGS#2, therefore, defines the patient population undergoing PGS#2 as distinctively different from earlier PGS#1 populations.

Even in older women and younger women with low ovarian reserve many more embryos survive in *in vitro* laboratory culture to day-3 after fertilization, where embryo biopsy was preformed for PGS#1, than to days 5/6, required for trophectoderm biopsy. Therefore, significantly more of such females would reach embryo transfer on day-3 than on days 5/6. Though disputed by some, there is, in fact, at least good anecdotal evidence that some embryos, which will not survive *in vitro* culture in the laboratory to days 5/6, if transferred on day-3 after fertilization, may still be able to establish normal pregnancies [12,15,16].

PGS#2 and PGS#1 outcomes can, and should, therefore, not be compared without statistically adjusting for difference in patient population. PGS#2 patients are clearly favorably selected in comparison.

(ii) Not only whether embryos survive to blastocyst stage in culture matters; how many survive matters as well. Older women and younger females with prematurely diminished ovarian reserve reach blastocyst stage with greatly diminished embryo numbers. Their risk of not reaching embryo transfer is, therefore, not only increased because so many of their embryos do not survive in the laboratory to days 5/6 but also because only comparatively few do survive to blastocyst stage. The fewer embryos a patient has available for trophectoderm biopsy and chromosomal evaluation, the higher the risk that all of the patient’s embryos will be aneuploid and, therefore, not available for embryo transfer.

Those patients who do reach embryo transfer in PGS#2 are, thus, automatically twice favorably selected.

This is a principal reason why all so far published PGS#2 studies, including those claiming outcome benefits for IVF, have been reporting outcomes in highly favorably selected patients. Only two so far published small studies have, however, pointed out this fact, one a small clinical trial of PGS#2 [17] and a small case control study, utilizing PGS#1, from our center [18]. That PGS#2, thus, automatically excludes significant patient populations, including older women who, in the initial utilization as PGS#1 represented the primary target population [4] is, therefore, neither communicated to the scientific community nor to patients receiving such treatments.

(iii) Finally, to define pregnancy outcomes for PGS#2 statistically correctly, it is essential to assess pregnancy rates by “intent to treat”. This means that pregnancy rates have to be calculated with denominator *cycle start* rather than *embryo transfer*. Yet, every single paper so far published in the literature, claiming any kind of outcome benefit for PGS#2, reported pregnancy outcomes with reference point embryo transfer. All of these studies, therefore, breach one of the most basic rules of statistical outcome reporting in IVF and, simply, misrepresent outcomes.

Misleading outcome reporting is, unfortunately, increasingly prevalent in the U.S., and has also contaminated federally mandated national IVF reporting [19]. Amongst a small number of U.S. clinics recently reported to artificially increase their reported live births rates we, as part of this investigation, indeed, noted a doubling of the number of PGS cycles during the 5-year study period to 8% of all center cycle activity, versus 4% for all other reporting clinics (Kushnir VA, Barad DH and Gleicher N, unpublished data).

(iv) While PGS#1 used fluorescence-in situ-hybridization (FISH) of a restricted number of chromosomes, likely the most profound advance in technology in PGS#2 is the ability to assess a complete chromosome complement via a 24-pair chromosome copy number analysis. Various companies and their respective assays now compete in the market place, with accuracy (i.e., false-positive and –negative rates) for most systems remaining to be determined. Some suggestions about efficacy can, however, be derived from a recently published study [20], though how individual testing platforms compare to each other also, still, needs to be established.

(v) Blastocyst stage in vitro culture has recently also been reported associated with two significant additional potential risks: In various small animal IVF models prolonged embryo culture has been demonstrated to lead to significant epigenetic changes, interfering with imprinting maintenance and DNA demethylation dynamics [21-23]. In addition, increasing human evidence suggests that blastocyst stage cultures are associated with increased premature delivery risk in comparison to earlier stage embryo transfers [24,25]. Combined, these observations raise further questions about PGS#2, which is dependent on blastocyst stage embryo biopsy.

### A study comparing day-3 and days 5/6 embryo biopsies

The study by Harton et al. [20] reported outcomes of PGS#2 for embryo biopsies performed on days-3 or days-5/6, utilizing the same newly developed array, comparative genomic hybridization technique (BlueGnome), which allows for analysis of a complete 24 chromosome complement on both biopsy days. This, therefore, is the first study comparing aneuploidy in a complete chromosome complement, performing either day-3 or days-5/6 embryo biopsies, utilizing one of the newly developed testing platforms.

While there are technical differences between these newly reported testing techniques, and comparative studies between different commercial platforms are not available, they share the claim that by allowing for assessments of a complete chromosome complement they are more accurate in determining embryo aneuploidy than the prior (exclusively on post-fertilization d-3) utilized FISH. Results of this study, in at least general terms, should therefore also be applicable to other reported platforms allowing for aneuploidy testing of a complete chromosome complement.

An analysis, comparing the same aneuploidy testing technique on days 3 and 5/6 after fertilization was overdue since the superiority in accuracy of aneuploidy diagnosis, claimed for these new diagnostic platforms appears compelling but has remained clinically unproven. To maintain its alleged superiority over PGS#1, days-5/6 trophectoderm biopsy, this key component of PGS#2, has to establish its superiority over day-3 embryo biopsy.

Recognizing the statistical and design weaknesses of this study, including lack of patient randomization to day-3 and days-5/6 biopsies, and unadjusted participation of multiple IVF centers with greatly varying IVF treatment protocols and patient populations, this study’s outcomes have to be interpreted with considerable caution. If data from this study are, however, considered “good enough” for proponents of PGS#2, fairness suggests that these data should also be available to support arguments of potential skeptics.

If the study is viewed in this way, it does offer potentially important new insights into efficacy of PGS#2. Those insights, however, in fact do not support efficacy of PGS#2 in improving IVF outcomes. A careful analysis of reported data, actually, offers further support for lack of therapeutic efficacy of PGS in general, and PGD#2 in particular.

Here is why: Reflected in the title of the paper, the main conclusion of the study was that, with utilization of their new array comparative genomic hybridization technique, up to age 42 years (and lesser extend above age 42), PGS#2 significantly diminished the effects of maternal age on embryo implantation and pregnancy rates. This conclusion was, however, once again based on implantation and pregnancy rates with reference embryo transfer rather than cycle start. In absence of an outcome analysis based on “intent-to-treat”, reported results, therefore, have to be viewed as statistically suspicious.

The authors also erred in concluding that widely reported declining rates of IVF success with advancing female age primarily have to be caused by aneuploidy since such an interpretation ignores that in women with poor ovarian reserve and/or small embryo numbers, embryo culture to days-5/6 blastocyst stage and/or embryo biopsy may have significantly contributed to their IVF failures [12,15].

The study does, however, offer some interesting additional findings: Aneuploidy rates were 21.4% higher (70-.6% vs. 49.2%) if embryos were biopsied on day-3, offering further evidence for a significant degree of self correction of embryos between days-3 and 5/6, as previously suggested [26], and often proposed as argument against day-3 embryo biopsies. Yet, implantation rates, even in those selected women who did reach embryo transfer, improved only by 9.6% (39.6% to 49.2%) in favor of days-5/6 embryo biopsies.

Since, as even the authors note in their manuscript, “some” women did not reach days-5/6 embryo transfer, the study raises the question whether the reported improvement in implantation rates between day-3 and days-5/6 biopsies would still be statistically significant if the outcome analysis had been performed by “intent-to-treat” (i.e. with reference cycle start).

The same question also arises in regards to presented data on pregnancy loss. Here, miscarriages after day-3 biopsy occurred in 9.9%, only 2.0% above the 7.9% for days-5/6 biopsies. Considering that almost a third of patients in both groups were above age 40 years old, both of these miscarriage rates appear unusually low. Analysis by “intent-to-treat” would, almost with certainty, not reveal a reduction in miscarriage rates for days-5/6 over day-3 biopsies.

Since pregnancy outcome data in the study are not presented in total, like implantation and miscarriage rates, but stratified by age groups, these data are somewhat difficult to interpret. They are also presented with two different reference points, per embryo biopsy (i.e., patients reaching embryo biopsy) and per embryo transfer (i.e., patients having at least 1 euploid embryo). Both reference points are, of course, removed from “intent to treat” since not every patient reaches embryo biopsy, and not every embryo reaching embryo biopsy will also be euploid and, therefore, transferrable.

The authors’ mode of data presentation, however, actually accentuates the importance of analysis by “intent to treat” since it well demonstrates that the reference point of embryo transfer is farthest removed from “intent to treat:” With reference point embryo biopsy, days-5/6 biopsies demonstrated significantly higher ongoing pregnancy rates than day-3 biopsies. Yet, even the authors noted that this statistical difference completely evaporated once comparisons were made with reference point embryo transfer, where days-5/6 biopsies no longer demonstrated outcome advantages over day-3 biopsies in terms of ongoing pregnancy rates.

This statistical observation, therefore, represents the most convincing evidence in the manuscript of Harton et al. that days-5/6 embryo biopsies do not appear to improve IVF outcomes in comparison to day-3 embryo biopsies. Even considering previously noted obvious methodical weaknesses, this study, therefore, offers rather convincing evidence that a major argument of PGS#2 proponents, almost with certainty, is inaccurate.

This, of course, raises further doubts about the hypothesis that PGS#2 represents a diagnostic clinical improvement over PGS#1, and that PGS#1 failed for technical reasons. The study by Harton et al., therefore, at minimum demonstrates that days-5/6 biopsies offer no outcome advantage over day-3 biopsies. Since, as noted earlier, embryo culture to blastocyst stage results in definite and significant clinical as well as cost disadvantages, the study by Harton et al., indeed, strongly suggests that, if PGS is to be performed at all as a tool of IVF outcome improvement, it actually should be performed on day-3 embryos, utilizing new diagnostic platforms for determination of aneuploidy in full chromosome complements.

### The repositioning of PGS#2 marketing

Since published studies have so far been unable to prove pregnancy outcome benefits for PGS#2, proponents of the procedure have started to promote the procedure for new indications. A prime example is the alleged PGD#2-driven ability of reducing twin pregnancies by facilitating embryo selection for elective single embryo transfer (eSET) [27].

Forman et al. recently reported that this represented the primary benefit of a clinical PGS#2 trial [27]. Their Clinical Trial Registration (#NCT011408433) at [27] notes, however, that the original primary intent of the study was improvement of IVF pregnancy rates. As this failed, their original intent was replaced by the listed secondary goal of the study, the reduction of twin pregnancies via eSET. We would argue that under generally accepted study reporting guidelines, such an unreported switch in study goals is inappropriate.

Specifically, the original Clinical Trial Registration lists as Primary Outcome Measures: (i) Live birth rate per randomized patient; and (ii) Comparative live birth rates of patients with elective single embryo transfer (eSET) of chromosomally normal embryos (after utilizing 24-chromosome copy analysis, given the acronym Comprehensive Chromosome Screening, CCS) and 2-embryo (2-ET) transfer without CCS. Only their Secondary Outcome Measures related to the risk of twinning. The published paper, however, does not refer to pregnancy rates in title of manuscript, and barely refers to pregnancy rates in the body of the manuscript.

The manuscript, however, describes itself in the title as a randomized controlled trial of single blastocyst stage embryo transfer, and it really is neither. The study design per initial registration (see above) was for a non-inferiority trial, demonstrating non-inferiority of transfer of a single embryo after PGS#2 in comparison to transfer of two embryos at blastocyst stage without trophectoderm biopsy and aneuploidy determination. Moreover, at 20% non-inferiority, the trial was set to demonstrate inferiority only if any difference between these two treatment arms exceeded 20%, a clinically potentially highly significant difference. In other words, even 19.9% inferiority in clinical outcome with transfer of a single euploid embryo would, still, have fallen within the excessive non-inferiority parameters set by the authors.

Pregnancy rates with single embryo transfer were, however, in absolute terms actually 4.4% lower and in relative terms 7.2% lower than with chromosomally untested double embryo transfer. Basically leaving this fact unaddressed in their manuscript, and claiming non-inferiority, the authors concentrated on above described secondary goal of their study, assessing in the literature already well-described effects of single embryo transfer on reducing twin pregnancy rates in association with IVF.

Reducing twin pregnancies represent a distinctively separate subject of considerable complexity from the primary goal of PGS#2 to improve IVF pregnancy and delivery rates. We, indeed, have extensively addressed the concept of single embryo transfer in the literature [28,29]. As a topic of further discussion, it is beyond the framework of this commentary. Only so much: Since the Clinical Trial Registry does not consider reduction of twin risks as the primary goal of the clinical trial, it appears unlikely that the patients’ primary intent (and informed consent) in participating in this trial was reduction of twinning risks. Much more likely, their participation was solicited with the intent of improving IVF pregnancy and delivery chances, a proposition infertility patients are supportive of [13].

As outcome reporting was, again, not based on “intent to treat”, reported IVF pregnancy rates are, in addition, inflated. Patients who did not have at least one euploid embryo for embryo transfer (in controls at least two blastocyst-stage embryos) were removed from outcome considerations. Consequently, 33/205 (16.1%) of original study participants were removed after the study’s initial selection criteria already excluded poor prognosis patients above age 42 years (AMH < 1.2 ng/mL and FSH > 12.0 IU/L). Selection biases in favor of favorable patients, thus, occurred in this study at three separate stages.

An analysis by “intent to treat”, would, therefore, likely further have lowered pregnancy rates by approximately 16.1%, resulting in pregnancy rates of 51.6% and 54.6%, respectively, for study and control groups, both rather unremarkable pregnancy rates for so highly selected, young patients. Indeed, these rates are lower than those reported by Schoolcraft’s group years ago in similarly selected women after blastocyst-stage eSET without any form of PGS [30].

As in many previously reported studies, single embryo transfer thus, even after PGS#2, resulted in lower IVF pregnancy rates than double-blastocyst stage embryo transfer with chromosomally unscreened embryos. Single embryo transfer, therefore, apparently will always reduce twin pregnancies but also always result in lower pregnancy rates than a 2-embryo transfer, raising significant questions about the utility of performing chromosomal analysis of embryos in even favorably selected women. At minimum, these data suggest that even favorably selected patients, still, require further selection to identify sub-populations who may benefit from PGS#2 [12,18]. Who these patients are (if such women, indeed, exist) remains, however, to be determined.

Even a suggestion that PGS#2 is responsible for the observed reduction in twin pregnancies in this study appears incorrect. Above noted study from Schoolcraft’s group claimed such a benefit from single embryo transfer already in 2004 [30]. Only a direct comparison of single embryo transfer with and without PGS#2 would allow for such a conclusion.

The study, in addition, did not meet even minimum criteria for a non-inferiority trial design. Interested readers on the subject of non-inferiority trials are referred to authoritative recent references, which in detail explain the complexities and requirements for such studies [31,32]. One also has to wonder about the purpose of a non-inferiority trial when the purpose of PGS is selection of euploid embryos to improve implantation and pregnancy rates [33]. Non-inferiority, therefore, becomes irrelevant; superiority should be the desired and investigated end point.

The paper by Forman et al., thus, paradoxically presents another very convincing clinical trial demonstrating that, even in highly favorably selected young women, PGS#2 appears ineffective in its primary goal, of improving IVF pregnancy rates.

## Conclusions

We spent extensive time commenting on the manuscripts by Harton et al. [20] and Forman et al. [27] because both of these manuscripts received, in our opinion, much too uncritical reception by the IVF community. The conclusions of the manuscript by Forman et al., to our surprise, indeed, not only received editorial support [14] but were even endorsed by the *American College of Obstetricians and Gynecologists (ACOG)*, which went to the extraordinary length of issuing a supportive opinion statement to the media [34].

This is especially surprising since policy statements, declaring PGS as ineffective in improving clinical pregnancy rates and reducing miscarriages, by the *American Society for Reproductive Medicine (ASRM)*[35], the *European Society for Human Reproduction and Embryology (ESHRE)*[36] and the *British Fertility Society*[37], issued after the failure of PGS#1 was recognized, still stand.

Mastenbroek et al., who were so crucial in reducing the worldwide utilization of PGS#1 [1], have since confirmed the ineffectiveness of PGS (largely PGS#1) in a systemic review and meta-analysis [38]. More recently, Mastenbroek also cautioned against the premature introduction of PGS#2 [39]. The recently published studies of Harton et al. [20] and Forman et al. [27] add further oil to the fire.

Considering that these authors, despite utilization of trophectoderm biopsy and state-of-the-arts aneuploidy testing, still, in even highly favorable patient populations, were unable to improve pregnancy rates suggests a quickly shrinking population base in which PGS may be effective. Indeed, one has to accept the increasing likelihood that the underlying paradigm for PGS, simply, may not work. The procedure just appears to increase costs and complexities of IVF. Its utilization, at present, should therefore be acknowledged as highly experimental and refuted in routine IVF care. Interestingly, Twisk et al. already in 2008 came to similar conclusions [7].

PGS#1 caused significant harm to thousands of patients, as noted before [5]. We should not allow it to happen again!

## Competing interests

All three authors have in the past received grant support for research, travel and speaking engagements, though none in any way related to topics discussed in this manuscript. N.G. and D.H.B. are listed as co-inventors on a number of already awarded and other still pending patents, none in any way related to in this paper discussed issues. N.G. is owner of CHR-New York, where this manuscript was produced, and owns shares in Fertility Nutraceuticals LLC. N.G. and D.H.B. receive royalty payments from Fertility Nutraceuticals LLC. V.A.K. consults to The Centers for Disease Control on matters of national ART outcome reporting.

## Authors’ contributions

All three authors considered a systematic review of the utilization of preimplantation genetic screening (PGS) in current clinical practice of highest importance, and contributed significantly at all stages of literature search, analysis of data and preparation of the manuscript. The primary literature search was performed, and the first manuscript draft was written by NG The other two authors then reviewed selected references, and significantly contributed to revisions of the manuscript before submission. All in the manuscript presented conclusions were reached unanimously, and all authors prior to submission approved the final manuscript.
